# Endovascular stent-induced alterations in host artery mechanical environments and their roles in stent restenosis and late thrombosis

**DOI:** 10.1093/rb/rby006

**Published:** 2018-05-02

**Authors:** Jinxuan Wang, Xuepu Jin, Yuhua Huang, Xiaolin Ran, Desha Luo, Dongchuan Yang, Dongyu Jia, Kang Zhang, Jianhua Tong, Xiaoyan Deng, Guixue Wang

**Affiliations:** 1 Key Laboratory of Biorheological Science and Technology, Ministry of Education; State and Local Joint Engineering Laboratory for Vascular Implants; Bioengineering College of Chongqing University, Chongqing, China; 2 Institute for Biomedical Engineering & Nano Science, Shanghai East Hospital, Tongji University School of Medicine, Shanghai, China; 3 Key Laboratory for Biomechanics and Mechanobiology of the Ministry of Education, School of Biological Science and Medical Engineering, Beihang University, Beijing, China

**Keywords:** stents, restenosis, hemodynamics, local mechanical environment

## Abstract

Cardiovascular stent restenosis remains a major challenge in interventional treatment of cardiovascular occlusive disease. Although the changes in arterial mechanical environment due to stent implantation are the main causes of the initiation of restenosis and thrombosis, the mechanisms that cause this initiation are still not fully understood. In this article, we reviewed the studies on the issue of stent-induced alterations in arterial mechanical environment and discussed their roles in stent restenosis and late thrombosis from three aspects: (i) the interaction of the stent with host blood vessel, involve the response of vascular wall, the mechanism of mechanical signal transmission, the process of re-endothelialization and late thrombosis; (ii) the changes of hemodynamics in the lumen of the vascular segment and (iii) the changes of mechanical microenvironment within the vascular segment wall due to stent implantation. This review has summarized and analyzed current work in order to better solve the two main problems after stent implantation, namely in stent restenosis and late thrombosis, meanwhile propose the deficiencies of current work for future reference.

## Introduction

Cardiovascular occlusive disease such as arteriosclerosis leads to poor blood circulation, angina pectoris, lack of oxygen and myocardial infarction. It has become one of the most threatening killers of human beings [1, 2]. Percutaneous coronary intervention is an effective and widely used therapy for atherosclerotic stenosis. Despite the effectiveness, this treatment faces late failure challenges resulting from in-stent restenosis (ISR) [3, 4] and thrombosis [5, 6]. According to clinical data, the restenosis rate of bare metal stents or drug-eluting stent (DES) can range from 16% to 44% or 3% to 20%, respectively [7]. Restenosis may occasionally cause fatal late thrombosis. To overcome the restenosis problem of bare metal stents, the DESs were developed, such as CD133-specific antibody-coated stents which have superiority in re-endothelialization and inhibition of ISR through promoting adhesion and proliferation of endothelial progenitor cells (EPCs), as well as rapamycin-coated stents, paclitaxel-coated stents and so on [8]. Unfortunately, mounting evidence has shown that the DES has two main problems, one is failed reduction of vascular damage, and another is the inhibited proliferation of smooth muscle cells (SMCs) and endothelium, leading to the formation of local thrombosis [9, 10]. However, at present, no drug can effectively restrain the growth of SMCs and promotes endothelial repair synchronously. At the same time, many researchers try to optimize the design of the scaffolds, using different flower patterns, thickness, and weaving mode to improve the performance of the scaffolds. Before solving these problems, we should first understand the interaction between the stent and the host blood vessels and the influence of the stent on the hemodynamics.

It has been well documented that vascular cells can adapt to mechanical stimuli, resulting in vascular remodeling [11]. Stent implantation significantly alters the mechanical environment within the host artery, possibly leading to the restenosis and thrombosis [5, 12]. To better understand the mechanism of ISR so that targeted strategies can be forged to prevent it, we must first clarify how the mechanical microenvironment within the host artery changes with time after a stent is deployed.

The goal of this review paper is to analyze the hemodynamics in the lumen after stent implantation, the mechanical microenvironment within the host artery, and to discuss their effects on re-endothelialization, restenosis, thrombosis formation and inflammation.

## Interactions between stents and host blood vessels

In this section, we will review the response of vascular wall and the mechanism of mechanical signal transmission, and then discuss the process of re-endothelialization and late thrombosis.

### Response of vascular wall after stent implantation

From inside to outside, there are three layers of the vascular wall: endothelial cells (ECs), SMCs and collagen fibers. ISR arises from multifactorial influences, including vascular intimal injury, thrombosis, vascular remodeling, inflammation and healing [13].

After stent implantation, the adventitia and medium will respond to the stent-induced alterations in the host artery mechanical environment, hence leading to the remodeling of the vessel wall and resulting in significant changes of its mechanical properties, such as the elastic modulus [14, 15]. In the case of vascular sclerosis and decreasing tensile stress, vascular SMCs (VSMCs) are switched into their synthetic state, which leads to the degradation of vascular matrix, more cell proliferation and migration, and ultimately vascular disease [16]. The elastic modulus of VSMCs basement is positively correlated with cell spreading projection area, and increased substrate elastic modulus promotes the proliferation and migration of VSMCs [17]. Peyton found that VSMCs generate greater stress on the substrates with high moduli when compared to the substrates with low moduli. The ligand concentration of the extracellular matrix (ECM) (especially fibronectin) is directly related to the number of migrating cells [18]. High levels of mechanical strain applied to fibronectin reduce the rates of both cell spreading and cell migration [19]. Muscle fibroblasts, which possess the characteristics of SMCs, have the ability to shrink, migrate and secrete ECM [20]. In blood vessels, fibroblasts, vascular ECs (VECs), VSMCs and monocytes on the scaffold can be differentiated into muscle fibroblasts [21]. Therefore, the increase in the elastic modulus of the blood vessels caused by the contractility of muscle fibroblasts and ECM large secretion may cause VSMCs hypertrophy and proliferation [22], leading to further fibrosis. Tissue fibrosis will result in decreased vascular compliance, increased elastic modulus, and the changes of SMCs and fibroblasts, which in turn change the biomechanical properties of the blood vessels.

**Figure 1:**
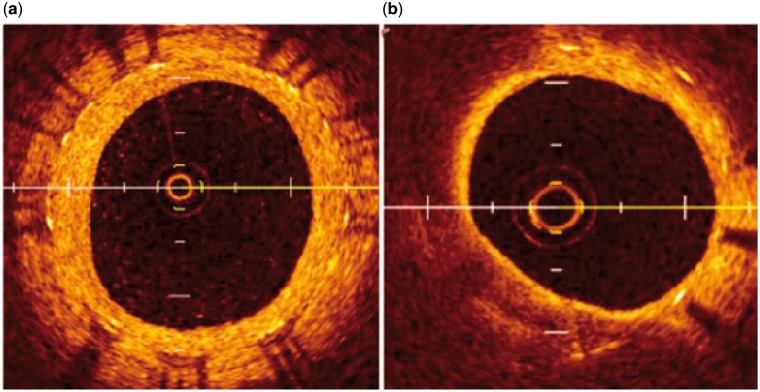
Optical coherence tomography images of common neointima and neoatherosclerosis. (a) Common neointima is recognized by its high-signal intensity and homogeneous region inside stent struts; (b) the neointima has a diffuse border and marked attenuation. Cited from [25], copyright 2015, with permission from *World J Cardiol*.

While atherosclerosis (AS) in native coronary arteries develops over decades, in-stent neoatherosclerosis seems to occur much faster, i.e. in months to years following stent placement [23]. It is speculated that incompetent and dysfunctional endothelial coverage of the stented segment contributes to this process. Stent implantation causes vascular injury with endothelial denudation. Incomplete maturation of the regenerated endothelium, which is characterized by poor cell-to-cell junctions, leads to reduced expression of antithrombotic molecules and low nitric oxide production. Pathologic intimal thickening with lipid pool is a hall mark of native AS and plaque progression, whereas in neoatherosclerosis necrotic core formation is mostly driven by macrophage apoptosis in the absence of lipid pool, which eventually leads to in-stent plaque rupture [24]. The normal neointima proliferates homogeneously, and the lipid-laden intima is not observed in the early phase. In the late phase, the lipid-laden intima is found in 67% of the cases (Fig. 1) [25].

Indeed, activated VSMCs could efficiently proliferate and migrate to contribute to vessel wall repair. However, the latest evidence [22] suggests that recruitment of rich hematopoietic stem cells/EPCs in the vessel wall during vascular remodeling, such as intimal hyperplasia and atherogenesis, allows SMCs to accumulate in the intima. Especially in chronic AS, arterial smooth muscle is abnormally regulated, resulting in enhanced SMC differentiation and ECM formation within the plaque region [26]. Therefore, it is generally accepted that the activation, migration, proliferation and further intimal hyperplasia of VSMCs are the main pathological bases for ISR [27].

### Response of ECs and the mechanism of mechanical signal transmission

**Figure 2:**
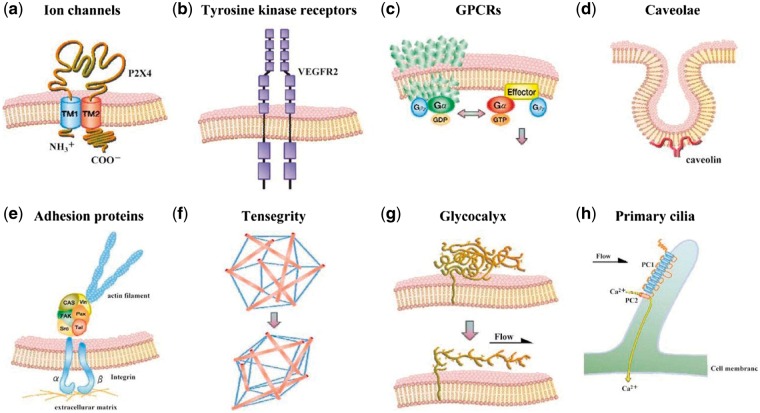
The mechanical receptors of VECs. (a) Ion channel; (b) tyrosine kinase receptor; (c) G-protein-coupled receptors; (d) cavelae; (e) adhesion molecule; (f) tensegrity; (g) glycocalyx and (h) primary cilium. Cited from [33], copyright 2009, with permission from C*irc J*.

**Figure 3:**
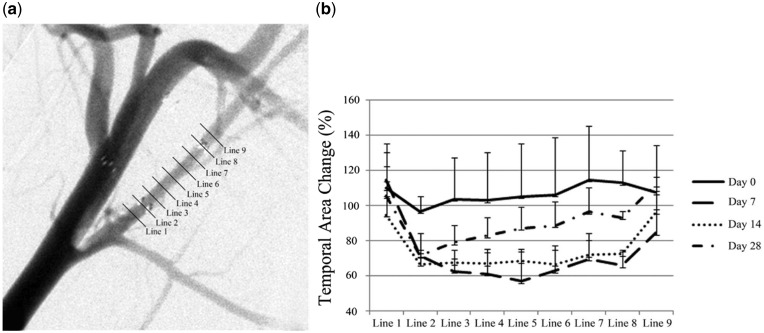
A characteristic pattern with time on the basis of the in-stent stenosis change. (a) Nine cross-sectional areas at 1.5-mm intervals, including the 1.5 mm proximal and distal to the stent; (b) temporal area change after wingspan stent placement, at days 0, 7, 14 and 28, was demonstrated in comparison with the preprocedural area. Cited from [31], copyright 2014, with permission from *Am J Neuroradiol*.

Stent implantation inevitably injures the ECs. Moreover, the stent-induced mechanical environmental changes can also lead to a series of changes in the ECs. ECs respond to shear stress via various mechanical sensitivity receptors, such as vascular endothelial growth factor receptor 2, VE-cadherin, platelet EC adhesion molecule-1, integrin, glycoprotein complex, primary cilium and glycocalyx (Fig. 2). Mechanical signals are transformed into biochemical information, which regulates the activation of multiple intracellular signaling pathways, such as inflammatory responses [28–30]. It is worth noting that the response of ECs to mechanical forces varies because of the size or direction of the force and the actual time fluctuation quantity. Laminar high shear stress (HSS) can affect cell polarity and protrusion of lamellipodia, and contraction of stress fibers by promoting cell actin cytoskeleton remodeling, thereby increasing migration of ECs. In contrast, low shear stress (LSS) is more likely to cause cell sloughing and assist cell migration due to larger shear stress gradient. The wall shear stress (WSS) demonstrates a characteristic pattern with time on the basis of the in-stent stenosis change (Fig. 3). The WSS gradient increases from the proximal to distal segment until day 14 after Wingspan stents placement. At day 28, the trend is reversed dramatically, decreasing from the proximal to the distal segment [31]. Shear stress regulates the cell cycle by activating Adenosine 5′-monophosphate (AMP)-activated protein kinase (AMPK) cascade reaction and the protein kinase B (PKB/AKT) signaling pathway [32]. Laminar HSS can simultaneously activate AMPK cascade and AKT signaling, maintain mammalian target of rapamycin (mTOR) in steady state to reduce the proliferation of ECs. However, low oscillatory shear stress only activates the AKT signaling pathway, but not the AMPK cascade, which leads to the proliferation of ECs through sustained activation of p70 ribosomal S6 kinase signaling molecules.

In addition to shear stress, because stent implantation alters the compliance of the host blood vessel, it will also affect the movement of the vascular wall under pulsatile blood pressure, hence the stretches of ECs. ECs have been confirmed to receive strain signal through the substantial cell membrane surface mechanical sensors, including cell adhesion sites [34], integrin receptors [35], tyrosine kinase receptor, ion channels [36] and lipid molecules [37], which transform mechanical signal into chemical signal, and further transmit it into the cell. The strain will activate transcription factors such as activator protein 1 and nuclear factor kappa-light-chain-enhancer of activated B cells, and regulate the expression of inflammatory genes. Meanwhile, the strain can induce the release of EC angiotensin-II, and activate angiotensin II type 1 receptor by up-regulating the expression of nicotinamide adenine dinucleotide phosphate (NADPH) oxidase peroxidase, which leads to the dysfunction of ECs and occurrence of inflammation [38].

### Re-endothelialization and late thrombosis

After stent implantation, thrombosis formed after 1 month to 1 year is defined as late thrombosis. Thrombosis formed after more than 1 year is defined as very late thrombosis [39]. The incidence of late stent thrombosis is only about 1%, but it causes more than 90% of the disability or death [40].

In the early stage after stent implantation, endothelial ablation, platelet aggregation and activation, coagulation cascade and strong inflammation locally happen in vascular. Endothelial layer is destroyed, and the stent surface is coated with a thin thrombotic layer. The main cellular component of the neointima is α-actin positive SMCs, which begin to migrate into the intima from the tunica elastica within several days after implantation. A healthy endothelial layer has tight junctions that can strictly regulate lipid and inflammatory molecule infiltration, and has balanced secretion of anticoagulant molecules that can provide good resistance to thrombosis formation [41]. Normal neointima with a smooth white membrane structure is antithrombogenic. On the other hand, the yellow neointima with neoarteriosclerosis can lead to thrombosis [25].

In the middle stage, ECs repair gradually completes, the coagulation cascade reaction decreases gradually, and SMCs migrate from the media membrane to the inner membrane [42]. Intimal thickening occurs and local inflammatory reaction tends to be normal. Studies have shown that vascular re-endothelializationafter injury can prevent metal stents from directly contacting the blood, which reduces flow disturbance and promotes the release of many active factors, such as VEGF, epidermal growth factor, platelet-derived growth factor (PDGF). This can effectively inhibit platelet activation/aggregation and thrombus formation in turn, finally reduce the incidence of ISR [43–45].

In the late stage, the re-endothelialization is completed but lacks entire function, because of the insufficient secretion of the anticoagulation factor. Therefore, late thrombosis may occur [46]. The re-endothelialization process mainly depends on the proliferation/migration of mature VECs derived from adjacent damaged intima, and on the homing, adhesion and differentiation of EPCs derived from bone marrow [47, 48]. Flow shear stress, tensile stress and the elastic modulus of the blood vessel can affect ECs migration [49–51]. The homing, proliferation and differentiation of endothelial stem cells (ESCs) can also be affected by flow shear stress [52]. Abnormal flow shear stress can lead to small plaque formation, thus restrain endothelial regeneration, hinder endothelialization and even cause endothelial dysfunction [53, 54]. HSS contributes to rupture-prone plaque formation through angiogenesis [55, 56]. This will eventually lead to late thrombosis. Therefore, the local hemodynamic environment plays an important role in the regulation of cell interaction and the regeneration of VECs, which affect the formation of thrombosis.

### Coronary artery calcification

Two recognized types of coronary artery calcification are atherosclerotic and medial artery calcification [57]. Vascular calcification is a hallmark of AS. The location, density and confluence of calcification may change portions of the arterial conduit to a noncompliant structure. Calcifications may also seed the cap of a thin cap fibro atheroma, altering tensile forces on the cap and rendering the lesion prone to rupture [58]. One effective method for the treatment of vascular calcification is stent implantation. However, the calcium deposits formed again over the years around the stent struts sometimes break into pieces, causing luminal thrombi [59–61]. This is likely due to a change in the local mechanical environment after stent implantation, contributing to more matrix vesicles produced by VSMCs matrix vesicles, and thereby regulates mineralization in the vascular intima and media. The effect of mechanical environment on the formation of vascular calcification needs further verification.

### Inflammation reaction

After stent implantation, the ECs are peeled off, and the subcutaneous tissue is exposed to the flowing blood. Although vascular mechanical damage caused by stent appears to be a prerequisite for the expression of the inflammatory response such as white blood cells and platelets, the inflammatory response and persistence may be related to individual factors. In particular, the increased expression of interleukin-antagonists (anti-inflammatory molecules) results in risk reduction of restenosis especially in young patients [62]. The reaction degree of ‘foreign matter’ triggered by the exposure of the strut is related to the persistence and sensitivity of individuals (see section 3.1, if you want to learn more about ‘strut’).

As early as 10–15 min after stent implantation, recruitment and aggregation of white blood cells can be detected in the stent area of the coronary artery [63, 64]. Infiltration of white blood cells, platelets and the change of the intimal lesion of the coronary artery result in a significant increase of the cell membrane surface. Platelet activation induces fibrinogen receptors, and leukocytes increase the expression of intercellular adhesion receptor factor [64, 65]. The stent can be covered with the neointima in 4 weeks. The rupture of the arterial tear and the plaque fibrous cap, and the support of the stent to the lipid core of the plaque can lead to enhanced inflammatory response, intimal hyperplasia, and a greater risk of ISR [64].

Recently, it has been shown that the main pathological basis of ISR is the activation, migration and proliferation of VSMCs with intimal hyperplasia [27]. Finding a drug that can effectively inhibit the proliferation of SMCs and the growth and migration of ECs, or developing a new kind of DES that can restrain the proliferation of smooth muscle and promote re-endothelialization may largely reduce the incidence of ISR.

## Stent-induced alteration in hemodynamics

**Figure 4:**
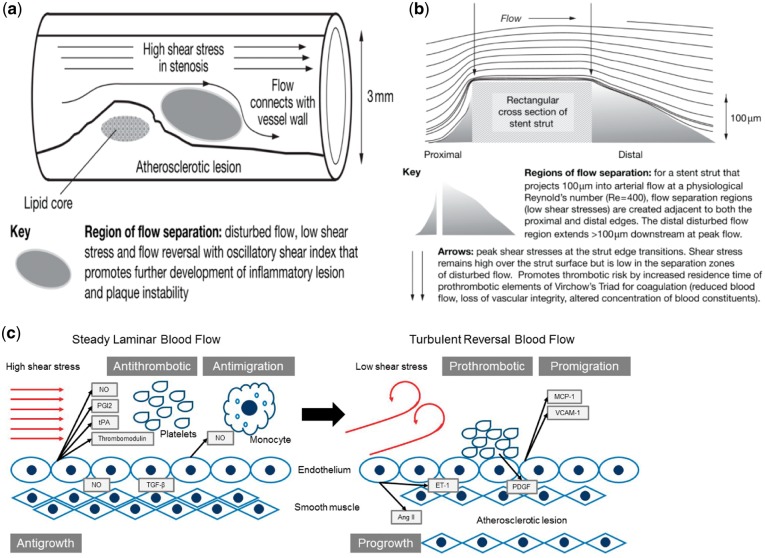
Flow separations at a stenosis (a) or a stent strut (b) that predispose to or contribute to pathogenesis. Cited from [74], copyright 2009, with permission from *Nat Clin Pr Cardiovasc Med*. (c) The influence of flow pattern and shear stress on the formation of AS.

In normal coronary arteries, blood flow takes a form of laminar flow, resulting in relatively high WSS on VECs. In the narrow section of the coronary artery with AS plaques, magnetic resonance imaging has been used to detect rapid increase in blood flow speed with significantly enhanced WSS [66]. Distal to the atherosclerotic stenosis, the WSS becomes relatively low due to the formation of flow separation and vortices (Fig. 4a) [66]. Histopathological changes in blood vessel wall, caused by either arteriosclerosis or thrombosis formation on the atherosclerotic lesions, will alter the geometry and the mechanical properties of the vessel, i.e. lumen narrowing, deformation and reduced elasticity of the vessel. These changes, in turn, lead to changes in hemorheological characteristics. For instance, when blood flow comes to the narrow section of the vessel, it may transit from laminar flow into turbulent flow depending on the degree of the stenosis, leading to abnormal HSS rate in the vicinity of the stenotic throat, but rather LSS in the vortex flow region distal to the stenosis. This is important because normal laminar flow shear stress is antithrombotic and antimigration, which can suppress atherogenesis. In contrast, disturbed flow with LSS is prothrombotic and promigration, which can lead to the genesis/development of AS (Fig. 4c) [67].

Stent implantation restores the stenotic lumen of the blood vessel to the normal state of dredging [49, 64–77], so that the HSS at the plaque stenosis will be reduced to the normal level with an improved local blood flow [78]. However, the stent implantation will inevitably cause some adverse hemodynamic changes in the lumen. For instance, the stent implanted will reduce the compliance of the vascular segment, leading to a compliance mismatch with other parts of the blood vessel. The compliance mismatch will affect the blood flow by creating flow separation zones. In addition, the intrusions of the stent struts (see section 3.1) into the lumen will also affect the blood flow resulting in small eddies with LSS, which is also pathogenic [66].

### Strut wire

**Figure 5:**
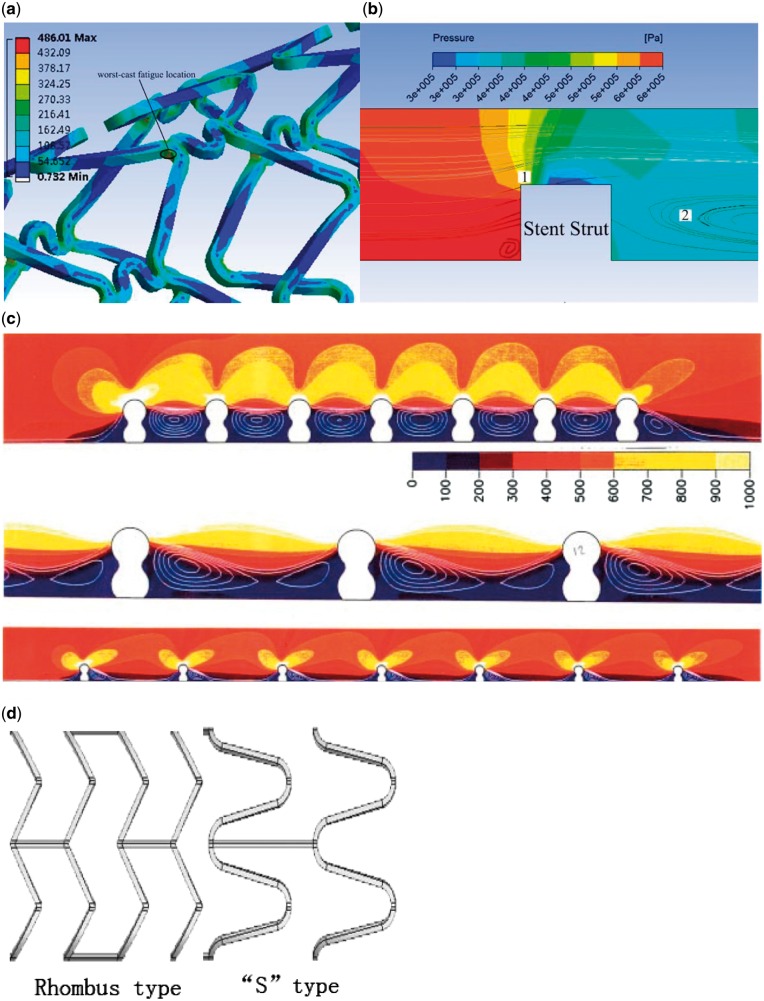
The influence factors of hemodynamic changes after stenting. (a) Finite element analysis of stress distribution after stent wire expansion. (b) Numerical simulation of the stress distribution around scaffold. (c) The influence of scaffold distance on hemodynamics, the color scales are adjusted to accentuate the flow patterns. Figure c cited from [91], copyright 2000, with permission from *Ann Biomed Eng.* (d) Stent strut of rhombus type and ‘s’ type.

Finite element analysis shows that the stress distribution of a stent after deployment is not uniform [79]. The major stressed areas occur at the locations where the struts deform the most, such as the bending segment [80–82]. Stress concentration may result in fracture of the struts. The worst fatigue most likely occurs along the lateral edge of a strut. The stress leads to the erosion of the coating (Fig. 5a) which leads to further degradation of stent effectiveness.

Currently, commercially available stents are manufactured using mesh-wired tube with certain thickness. As mentioned previously, the thickness of the strut leads to the formation of vortices or disturbed flow. On one hand, disturbed flow with vortices may facilitate deposition of substances. On the other hand, due to the intrusion of the stent into the vascular lumen, different stress distribution of the proximal and distal end of the stent leads to changes in hemodynamics. As shown in Fig. 5b, the high shear force in zone 1 can activate platelets, the activated platelet then will be trapped in zone 2, leading to increased concentration of cell factors such as PDGF (Fig. 5b).

### Strut space

Different strut spaces lead to different stress distribution both at the proximal and distal end of the strut [83]. In a stent with short strut space, flow stagnation region between adjacent struts will overlap, which is not the case for a stent with long strut space (Fig. 5c).

Compared with ‘rhombus’ stent, the ‘S’ type stent has lower radial stiffness, which results in less stress magnitude and radial displacement of the arterial wall (Fig. 5c) [84]. In particular, strut thickness and stent flexibility have been recognized to impact the degree of injury, risk of rupture of the elastic laminae and overall inflammation, with a thicker strut generally leading to higher inflammation and restenosis than the thinner one and open cell design [85]. Thus, we believe that the flower pattern of stent significantly changes the flow direction, and further affects the alteration in hemodynamic. Moreover, such a change certainly affects cellular functions. However, to verify this statement, additional investigations are needed.

### Compliance changes

Stent implantation changes the compliance of the blood vessel, which also affects the hemodynamics in the blood vessel [86]. Stiff metal struts compromise the geometry of the host artery leading to long-term adverse hemodynamic conditions and chronic stimulation, which further cause restenosis and clinical events [87–89]. For instance, stent implantation not only alters the 3D configuration of the host artery, but also alters the curvatures of its inlet and outlet, which lead to the changes in local shear stress distribution [90]. To a certain extent, these changes may explain the asymmetric mode of the restenosis in scaffold.

Adverse hemodynamic alteration by stent implantation is only one of the major factors leading to ISR. Since other forces can also affect the surrounding mechanical environment of vascular cells, the mechanism of the cellular response to its surrounding mechanical environment alterations needs further studies. Furthermore, the alteration of mechanical environment after stent implantation may postpone re-endothelialization that further leads to late thrombosis formation. However, the vascular cells may not directly respond to the force from external environment, perhaps in an indirect way or other signals. Therefore, how to optimize the stent design to reduce the degree of changes in vascular internal mechanical environment is a problem for us to consider, such as trying different kinds of flower patterns and softer scaffold materials.

## Mechanical microenvironment of stent vascular segment

In this section, we will review the changes in the mechanical microenvironment of the host artery due to stent implantation and the effects of these changes on vascular cells in an attempt to better understand the mechanism of ISR and late thrombosis formation.

**Figure 6:**
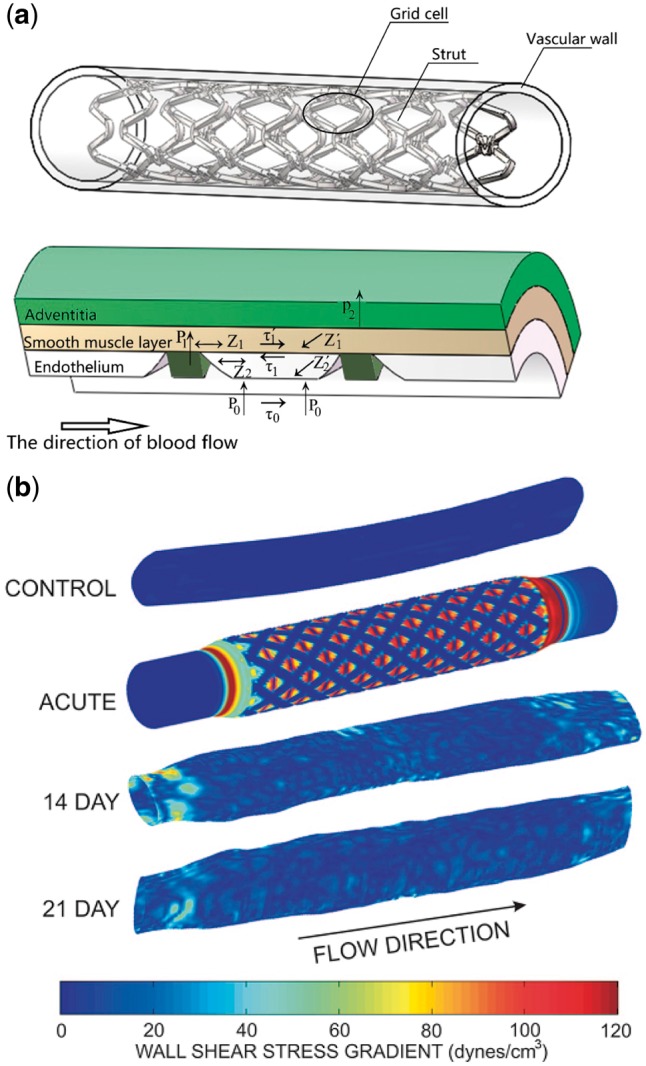
Mechanical environment of vascular tissue near the scaffold. (a) After expansion, stent filaments will cause damage to vascular endothelial, then reach the smooth muscle layer through endothelium, adjacent stent wires come about radial extrusion P1 and P2 on smooth muscle layer and cortex, respectively, axial tension Z1 and circumferential tension Z′1 on smooth muscle layer, as well as axial tension Z2 and circumferential tension Z′2 on endothelium. Due to varying extension of smooth muscle layer and endothelium, on the interface of which will come about shear stress *τ*1 and *τ*′1, meanwhile, this part of the endothelium will face the blood pressure P0 and WSS *τ*0 under the blood flow. (b) Spatial WSS gradient with time after stent implantation. Figure b cited from [73], copyright 2002, with permission from *Ann Biomed Eng*.

The data show a clear impact of the vascular mechanical microenvironment on vessel cells. Figure 6a is a schematic drawing of different stresses acting on the host artery by a single stent scaffold, showing a clear impact of the vascular mechanical microenvironment on vessel cells. As shown in Fig. 6, after stent implantation, ECs of the intima and SMCs of the medium experience pressure stress, tension stress, and flow-induced shear stress [69]. From this viewpoint, ISR actually is a healing process of the arterial wall after injury due to squeezing pressure by the stent wires. This process is composed of three main steps: elastic recoil, vascular intimal hyperplasia and vascular remodeling. The cells in the adventitia and medium can sense the mechanical changes in their surroundings (mechanical environment) during the stenosis process accordingly conduct mechanical signals and adjust their responses. For example, the stent diameter can directly affect the circumferential stress (Z′1, Z′2) of the artery that has an obvious impact on the intimal hyperplasia process. Both too large and too small diameters of the stent can significantly increase the degree of intimal hyperplasia [70]. The axial tension (Z1, Z2) of the stent can also affect intimal hyperplasia [71]. The stent will interfere with local flow altering the distribution of flow-induced WSS (*τ*_0_) [54]. The number, shape, thickness and width of stent struts, the launched diameter and the weaving mode of the stent can significantly affect the temporal and spatial distribution of local flow patterns, hence the flow-induced WSS [47]. Both animal experiments and clinical studies have found that the flow shear stress is related to the degree of intimal hyperplasia induced by vascular stent [72].

Numerical simulation analysis of the thrombus-induced spatial WSS gradient with time suggests that stents cause a transient HSS gradient after implantation and that the shear stress gradually returns to normal 14 days after implantation (Fig. 6b) [73].

Although it has been recognized that ISR and late thrombosis are mainly attributed to stent-induced mechanical microenvironment alterations, the regularity of the stent-induced mechanical changes in the host artery is still far from clarified because most of the existing studies regarding this aspect are based on very simplified models, which cannot reflect the real dynamic scenarios of the host artery with stent implantation. The future work can be based on the changes in the mechanical microenvironment after the stent implantation, which can affect the mediator and thus cause the ISR. Improving evaluation means of mechanical microenvironmental changes caused by the design of stent is conducted to better serve the stent design and optimize the mechanical environment of vascular stent.

## Conclusion and future perspective

It has been widely documented that ISR is mostly attributed to the changes in mechanical microenvironment due to stent implantation [5, 12]. Without in-depth understanding of these mechanical changes regularity, it would be impossible to find targeted/effective ways to resolve the two major problems of stenting, namely the restenosis and late thrombosis. This is precisely what is lacking in the existing literature. In the present article, we have summarized what has been done up to now regarding this issue in a hope to attract the attention of scholars.

ISR is a repair process of the arterial wall after mechanical injury, consisting of elastic retraction, intimal hyperplasia and vascular remodeling [73]. In this process, vascular cells such as SMCs and fibroblasts respond to stent-induced mechanical environment changes by adjusting their phonotype and behavior. Under the condition of angiosclerosis and reduced tensile stress, SMCs not only change their phonotype from the contractile state to the synthetic state, leading to vascular matrix degradation, but also proliferate or migrate, causing the genesis or development of vascular lesions, respectively [16]. In addition, the basal elastic modulus of SMCs shows a positive correlation with spreading projection area. Increased basal elastic modulus can promote SMCs proliferation or migration, which has a positive correlation with ligand concentration of the ECM (especially fibronectin) [18]. Myofibroblast is one type of fibroblasts with SMCs-like characteristics, which has strong ability to contract, migrate and secrete ECM [20]. Not only fibroblasts, ECs and SMCs can differentiate into myofibroblast [21]. The increase in elastic modulus caused by vasoconstriction, and a large amount of ECM secreted by fibroblasts, causing SMCs hypertrophy and proliferation [22], resulting in further vascular fibrosis. Unfortunately, the occurrence time of muscle fibroblasts appearing and apoptosis after stent implantation do not have the systematically thorough research. After stent implantation, effective inhibition of the proliferation and migration of SMCs may delay the progression of vascular fibrosis and thus reduce the incidence of ISR.

The delayed healing of EC layer after stent implantation is considered to be the key pathological event leading to late stent thrombosis. It is believed that the proliferation and migration of ECs in uninjured artery segment might eventually repair the wounded EC layer [92], but this process could be influenced by flow shear stress and tensile stress [49], and also by vascular elastic modulus [50]. Obviously, stent implantation has significant adverse impacts on these mechanical parameters, which hinder EC migration and interrupts bone marrow-derived ESCs homing, proliferation and differentiation. Moreover, stent implantation–induced local flow disturbance itself can cause small plaque formation that slows down endothelium healing and even causes endothelial dysfunction [53, 54]. Therefore, stent implantation may inevitably create an unfavorable mechanical environment for late thrombosis formation. Many drugs that can availably promote endothelialization have achieved a certain effect, but endothelial dysfunction and hemodynamic environment changes in stented segment are still serious problems to be solved, which put forward higher requirements of endothelial growth promoting drug screening and rational design of the stent.

The immune cells in blood are regulated by flow shear stress [93]. For instance, the migration and activation of lymphocytes are regulated by shear stress via the integrin dependent pathway [94]. A large number of EPCs, dendritic cells and neural crest derived cells are found in the vascular neointima after stent implantation, but nearly none in the media layer [95]. Therefore, it has been believed that these three kinds of cells might play important roles in the formation of new endothelial layer [96–98]. However, the role of the interaction between ECs and dendritic cells in VSMCs and mechanism of thrombosis formation induced by it under the conditions of hemodynamic changes has not yet been elucidated.

To sum up, finding out how the vascular stent implantation changes the biomechanical environment of blood vessels, thereby affecting the formation of restenosis and late thrombosis, and its molecular mechanism is a scientific problem of great concern. Based on this, future researches can be carried out from the following three aspects. Presently, most of the work on this issue is based on simplified theoretical models, lacking *in vivo* experimental verification. Theoretical models of the blood vessel and the stent after stent implantation can be predicted by numerical simulation, but the interaction between the stent and the blood vessel, blood vessel and blood cannot be directly reflected. In the future, more realistic numerical models are needed to elucidate the dynamic interaction between the stent and the host artery in terms of hemodynamic and vessel WSS distributions including the distributions of flow shear stress, the axial, radial and circumferential stress of vascular wall. Then, based on the detailed information of stent-induced mechanical environment changes obtained, systematic study should be designed to clarify the responses of the vascular and vascular immune cells to these mechanical stimuli, so as to build a more realistic model where we could get more hypotheses. The model can be improved when the new hypothesis is verified. Second, systematically study the effects of mechanical environment changes on the physiological characteristics of vascular cells (ECs, SMCs and fibroblasts), cells (mainly dendritic cells) that are involved in vascular immune system, and vascular EPCs that are involved in vascular injury repair. Finally, systematically clarify the interactive relationship between mechanic-cell biological behavior and its biological significance in ISR, and the relationship between mechanic-vascular biological behaviors, explore the molecular mechanism involved in the ISR and re-endothelialization. In this way, we may find targeted and effective ways to resolve the two problems of stenting.

## References

[1] Cregler LL. Cocaine: the newest risk factor for cardiovascular disease. Clin Cardiol 1991;14:449–56. 1810680 10.1002/clc.4960140626

[2] Sanchis-Gomar F, Perez-Quilis C, Leischik R et al Epidemiology of coronary heart disease and acute coronary syndrome. Ann Transl Med 2016;4:256. 27500157 10.21037/atm.2016.06.33 PMC4958723

[3] Byrne RA, Joner M, Kastrati A. Stent thrombosis and restenosis: what have we learned and where are we going? Eur Heart J 2015;36:3320–31. 26417060 10.1093/eurheartj/ehv511 PMC4677274

[4] Siontis GC, Stefanini GG, Mavridis D et al Percutaneous coronary interventional strategies for treatment of in-stent restenosis: a network meta-analysis. Lancet 2015;386:655–64. 26334160 10.1016/S0140-6736(15)60657-2

[5] Charonko J, Karri S, Schmieg J et al In vitro comparison of the effect of stent configuration on wall shear stress using time-resolved particle image velocimetry. Ann Biomed Eng 2010;38:889–902. 20099035 10.1007/s10439-010-9915-7

[6] Hofma SH, Brouwer J, Velders MA et al Second-generation everolimus-eluting stents versus first-generation sirolimus-eluting stents in acute myocardial infarction: 1-year results of the randomized XAMI (XienceV Stent vs. Cypher Stent in Primary PCI for acute myocardial infarction) trial. J Am Coll Cardiol 2012;60:381–7. 22835668 10.1016/j.jacc.2012.01.073

[7] Alraies MC, Darmoch F, Tummala R et al Diagnosis and management challenges of in-stent restenosis in coronary arteries. World J Cardiol 2017;9:640–51. 28932353 10.4330/wjc.v9.i8.640 PMC5583537

[8] Wu X, Yin TY, Tian J et al Distinctive effects of CD34- and CD133-specificantibody-coatedstents on re-endothelializationand in-stent restenosis at the early phase ofvascular injury. Regen Biomater 2015;2:87–96. 26813006 10.1093/rb/rbv007 PMC4669017

[9] Curfman GD, Morrissey S, Jarcho JA et al Drug-eluting coronary stents - Promise and uncertainty. N Engl J Med 2007;356:1059–60. 17296828 10.1056/NEJMe068306

[10] Prophylaxis A, Gray GE, Ch B et al (n.d.). edi t or i a l, 8–10.

[11] Chatterjee S, Fujiwara K, Pérez NG et al Mechanosignaling in the vasculature: emerging concepts in sensing, transduction and physiological responses. Am J Physiol Hear Circ Physiol 2015;308:H1451–62. 10.1152/ajpheart.00105.2015 PMC4469874 25862828

[12] Korshunov VA, Schwartz SM, Berk BC. Vascular remodeling: hemodynamic and biochemical mechanisms underlying Glagov’s phenomenon. Arterioscler Thromb Vasc Biol 2007;27:1722–8. 17541029 10.1161/ATVBAHA.106.129254

[13] Schiele TM. Current understanding of coronary in-stent restenosis: pathophysiology, clinical presentation, diagnostic work-up, and management. Z Kardiol 2005;94:772–90. 16258781 10.1007/s00392-005-0299-x

[14] Lal BK, Hobson RW, Goldstein J et al Carotid artery stenting: is there a need to revise ultrasound velocity criteria? J Vasc Surg 2004;39:58–66. 14718815 10.1016/j.jvs.2003.10.043

[15] Dobson G, Flewitt J, Tyberg JV et al Endografting of the descending thoracic aorta increases ascending aortic input impedance and attenuates pressure transmission in dogs. Eur J Vasc Endovasc Surg 2006;32:129–35. 16564712 10.1016/j.ejvs.2006.01.020

[16] Gambillara V, Thacher T, Silacci P et al Effects of reduced cyclic stretch on vascular smooth muscle cell function of pig carotids perfused ex vivo. Am J Hypertens 2008;21:425–31. 18219296 10.1038/ajh.2007.72

[17] Sazonova OV, Lee KL, Isenberg BC et al Cell-cell interactions mediate the response of vascular smooth muscle cells to substrate stiffness. Biophys J 2011;101:622–30. 21806930 10.1016/j.bpj.2011.06.051 PMC3145282

[18] Peyton SR, Putnam AJ. Extracellular matrix rigidity governs smooth muscle cell motility in a biphasic fashion. J Cell Physiol 2005;204:198–209. 15669099 10.1002/jcp.20274

[19] Hubbard B, Buczek-Thomas JA, Nugent MA et al Fibronectin fiber extension decreases cell spreading and migration. J Cell Physiol 2016;231:1728–36. 26621030 10.1002/jcp.25271

[20] Forte A, Della Corte A, De Feo M et al Role of myofibroblasts in vascular remodelling: focus on restenosis and aneurysm. Cardiovasc Res 2010;88:395–405. 20621923 10.1093/cvr/cvq224

[21] Stewart HJS, Guildford AL, Lawrence-Watt DJ et al Substrate-induced phenotypical change of monocytes/macrophages into myofibroblast-like cells: a new insight into the mechanism of in-stent restenosis. J Biomed Mater Res Part A 2009;90A:465–71. 10.1002/jbm.a.32100 18546184

[22] Glasser SP, Arnett DK, McVeigh GE et al Vascular compliance and cardiovascular disease – A risk factor or a marker? Am J Hypertens 1997;10:1175–89. 9370391 10.1016/s0895-7061(97)00311-7

[23] Otsuka F, Sakakura K, Yahagi K et al Has our understanding of calcification in human coronary atherosclerosis progressed? Arterioscler Thromb Vasc Biol 2014;34:724–36. 24558104 10.1161/ATVBAHA.113.302642 PMC4095985

[24] Otsuka F, Byrne RA, Yahagi K et al Neoatherosclerosis: overview of histopathologic findings and implications for intravascular imaging assessment. Eur Heart J 2015;36:2147–59. 25994755 10.1093/eurheartj/ehv205

[25] Komiyama H, Takano M, Hata N et al Neoatherosclerosis: coronary stents seal atherosclerotic lesions but result in making a new problem of atherosclerosis. World J Cardiol 2015;7:776–83. 26635925 10.4330/wjc.v7.i11.776 PMC4660472

[26] Chistiakov DA, Orekhov AN, Bobryshev YV. Vascular smooth muscle cell in atherosclerosis. Acta Physiol 2015;214:33–50. 10.1111/apha.12466 25677529

[27] Wu X, Liu W, Jiang H et al Kindlin-2 siRNA inhibits vascular smooth muscle cell proliferation, migration and intimal hyperplasia via Wnt signaling. Int J Mol Med 2016;37:436–44. 26676966 10.3892/ijmm.2015.2429

[28] Li Y, Bhindi R, Khachigian LM. Recent developments in drug-eluting stents. J Mol Med (Berl) 2011;89:545–53. 21279500 10.1007/s00109-011-0729-3

[29] Lin YM, Li F, Shi XZ. Mechanical stress is a pro-inflammatory stimulus in the gut: in vitro, in vivo and ex vivo evidence. PLoS One 2014;9:e106242. 25180799 10.1371/journal.pone.0106242 PMC4152012

[30] Manuyakorn W, Smart DE, Noto A et al Mechanical strain causes adaptive change in bronchial fibroblasts enhancing profibrotic and inflammatory responses. PLoS One 2016;11:e0153926. 27101406 10.1371/journal.pone.0153926 PMC4839664

[31] Fujimoto M, Takao H, Suzuki T et al Temporal correlation between wall shear stress and in-stent stenosis after Wingspan stent in swine model. Am J Neuroradiol 2014;35:994–8. 24231853 10.3174/ajnr.A3773 PMC7964559

[32] Zhao L, Fan C, Zhang Y et al Adiponectin enhances bone marrow mesenchymal stem cell resistance to flow shear stress through AMP-activated protein kinase signaling. Sci Rep 2016;6:28752. 27418435 10.1038/srep28752 PMC4945870

[33] Ando J, Yamamoto K. Vascular mechanobiology: endothelial cell responses to fluid shear stress. Circ J 2009;73:1983–92. 19801852 10.1253/circj.cj-09-0583

[34] Shay-Salit A, Shushy M, Wolfovitz E et al VEGF receptor 2 and the adherens junction as a mechanical transducer in vascular endothelial cells. Proc Natl Acad Sci U S A 2002;99:9462–7. 12080144 10.1073/pnas.142224299 PMC123163

[35] Sun X, Fu Y, Gu M et al Activation of integrin α5 mediated by flow requires its translocation to membrane lipid rafts in vascular endothelial cells. Proc Natl Acad Sci U S A 2016;113:769–74. 26733684 10.1073/pnas.1524523113 PMC4725528

[36] D’hondt C, Himpens B, Bultynck G. Mechanical stimulation-induced calcium wave propagation in cell monolayers: the example of bovine corneal endothelial cells. J Vis Exp 2013;e50443. 23892350 10.3791/50443 PMC3805061

[37] Lee CL, Moding EJ, Cuneo KC et al p53 Functions in endothelial cells to prevent radiation-induced myocardial injury in mice. Sci Signal 2012;5:ra52. 22827996 10.1126/scisignal.2002918 PMC3533440

[38] Wheeler-Jones CP, Farrar CE, Pitsillides AA. Targeting hyaluronan of the endothelial glycocalyx for therapeutic intervention. Curr Opin Investig Drugs 2010;11:997–1006. 20730694

[39] Jaffe R, Strauss BH. Late and very late thrombosis of drug-eluting stents. Evolving concepts and perspectives. J Am Coll Cardiol 2007;50:119–27. 17616295 10.1016/j.jacc.2007.04.031

[40] Stone GW, Ellis SG, Colombo A et al Offsetting impact of thrombosis and restenosis on the occurrence of death and myocardial infarction after paclitaxel-eluting and bare metal stent implantation. Circulation 2007;115:2842–7. 17515458 10.1161/CIRCULATIONAHA.106.687186

[41] Wu KK, Thiagarajan P. Role of endothelium in thrombosis and hemostasis. Annu Rev Med 1996;47:315–31. 8712785 10.1146/annurev.med.47.1.315

[42] Silverman-Gavrila R, Silverman-Gavrila L, Hou G et al Rear polarization of the microtubule-organizing center in neointimal smooth muscle cells depends on PKCα, ARPC5, and RHAMM. Am J Pathol 2011;178:895–910. 21281821 10.1016/j.ajpath.2010.10.001 PMC3128507

[43] Sharif F, Hynes SO, Cooney R et al Gene-eluting stents: adenovirus-mediated delivery of eNOS to the blood vessel wall accelerates re-endothelialization and inhibits restenosis. Mol Ther J Am Soc Gene Therapy 2008;16:1674. 10.1038/mt.2008.165 18714308

[44] Bedair TM, Elnaggar MA, Joung YK et al Recent advances to accelerate re-endothelialization for vascular stents. J Tissue Eng 2017;8:2041731417731546. 28989698 10.1177/2041731417731546 PMC5624345

[45] Xia CY, Yu AX, Qi B et al Analysis of blood flow and local expression of angiogenesis-associated growth factors in infected wounds treated with negative pressure wound therapy. Mol Med Rep 2014;9:1749–54. 24584462 10.3892/mmr.2014.1997

[46] Joner M, Nakazawa G, Finn AV et al Endothelial cell recovery between comparator polymer-based drug-eluting stents. J Am Coll Cardiol 2008;52:333–42. 18652940 10.1016/j.jacc.2008.04.030

[47] Rie KY, Ii M, Masuo O et al Cilostazol activates function of bone marrow-derived endothelial progenitor cell for re-endothelialization in a carotid balloon injury model. PLoS One 2011;6:e24646. 21931795 10.1371/journal.pone.0024646 PMC3171459

[48] Wara AK, Manica A, Marchini JF et al Bone marrow-derived kruppel-like factor 10 controls reendothelialization in response to arterial injury. Arterioscler Thromb Vasc Biol 2013;33:1552–60. 23685559 10.1161/ATVBAHA.112.300655 PMC3835145

[49] Chien S, Li S, Shiu YT et al Molecular basis of mechanical modulation of endothelial cell migration. Front Biosci 2005;10:1985–2000. 15769679 10.2741/1673

[50] Correia ML, Haynes WG. Arterial compliance and endothelial function. Curr Diab Rep 2007;7:269–75. 17686402 10.1007/s11892-007-0043-1

[51] Balcells M, Martorell J, Olivé C et al Smooth muscle cells orchestrate the endothelial cell response to flow and injury. Circulation 2010;121:2192–9. 20458015 10.1161/CIRCULATIONAHA.109.877282 PMC2887340

[52] Zeng L, Xiao Q, Margariti A et al HDAC3 is crucial in shear- and VEGF-induced stem cell differentiation toward endothelial cells. J Cell Biol 2006;174:1059–69. 16982804 10.1083/jcb.200605113 PMC2064396

[53] Duraiswamy N, Jayachandran B, Byrne J et al Spatial distribution of platelet deposition in stented arterial models under physiologic flow. Ann Biomed Eng 2005;33:1767–77. 16389525 10.1007/s10439-005-7598-2

[54] Chiu JJ, Chien S. Effects of disturbed flow on vascular endothelium: pathophysiological basis and clinical perspectives. Physiol Rev 2011;91:327–87. 21248169 10.1152/physrev.00047.2009 PMC3844671

[55] Qiu JH, Lei DX, Hu JJ et al Effect of intraplaque angiogenesis toatherosclerotic rupture-prone plaque induced byhigh shear stress in rabbit model. Regen Biomater 2017;4:215–22. 28798867 10.1093/rb/rbx007 PMC5544912

[56] Wang Y, Qiu JH, Luo SS et al High shear stress induces atheroscleroticvulnerable plaque formation throughangiogenesis. Regen Biomater 2016;3:257–67. 27482467 10.1093/rb/rbw021 PMC4966293

[57] Madhavan MV, Tarigopula M, Mintz GS et al Coronary artery calcification: pathogenesis and prognostic implications. J Am College Cardiol 2014;63:1703–14. 10.1016/j.jacc.2014.01.017 24530667

[58] Nakahara T, Dweck MR, Narula N et al Coronary artery calcification from mechanism to molecular imaging. J Am Coll Cardiol Img 2017;10:582–93. 10.1016/j.jcmg.2017.03.005 28473100

[59] Khattab AA, Otto A, Hochadel M et al Drug-eluting stents versus bare metal stents following rotational atherectomy for heavily calcified coronary lesions: late angiographic and clinical follow-up results. J Interv Cardiol 2007;20:100–6. 17391217 10.1111/j.1540-8183.2007.00243.x

[60] Clavijo LC, Steinberg DH, Torguson R et al Sirolimus-eluting stents and calcified coronary lesions: clinical outcomes of patients treated with and without rotational atherectomy. Cathet Cardiovasc Intervent 2006;68:873–8. 10.1002/ccd.20615 17086529

[61] Furuichi S, Sangiorgi GM, Godino C et al Rotational atherectomy followed by drug-eluting stent implantation in calcified coronary lesions. EuroIntervention 2009;5:370–4. 19736163 10.4244/v5i3a58

[62] Welt FGP, Rogers C. Inflammation and restenosis in the stent era. Arterioscler Thromb Vasc Biol 2002;22:1769–76. 12426203 10.1161/01.atv.0000037100.44766.5b

[63] Kornowski R, Hong MK, Tio FO et al In-stent restenosis: contributions of inflammatory responses and arterial injury to neointimal hyperplasia. J Am Coll Cardiol 1998;31:224–30. 9426044 10.1016/s0735-1097(97)00450-6

[64] Kastrati a, Koch W, Berger PB et al Protective role against restenosis from an interleukin-1 receptor antagonist gene polymorphism in patients treated with coronary stenting. J Am Coll Cardiol 2000;36:2168–73. 11127457 10.1016/s0735-1097(00)01014-7

[65] Inoue T, Croce K, Morooka T et al Vascular Inflammation and Repair: implications for Reendothelialization, Restenosis, and Stent Thrombosis. JACC Cardiovasc Intervent 2011;4:1057–66. 10.1016/j.jcin.2011.05.025 PMC3341937 22017929

[66] Davies PF. Hemodynamic shear stress and the endothelium in cardiovascular pathophysiology. Nat Clin Pr Cardiovasc Med 2009;6:16–26. 10.1038/ncpcardio1397 PMC2851404 19029993

[67] Tsuchiya K, Kirima K, Yoshizumi M et al New methods to evaluate endothelial function: evaluation of endothelial function by hemoglobin-nitric oxide complex using electron paramagnetic resonance spectroscopy. J Pharmacol Sci 2003;93:417–22. 14737011 10.1254/jphs.93.417

[68] Tanaka H, Sukhova GK, Swanson SJ et al Sustained activation of vascular cells and leukocytes in the rabbit aorta after balloon injury. Circulation 1993;88:1788–803. 7691431 10.1161/01.cir.88.4.1788

[69] Haga JH, Li Y-SJ, Chien S. Molecular basis of the effects of mechanical stretch on vascular smooth muscle cells. J Biomech 2007;40:947–60. 16867303 10.1016/j.jbiomech.2006.04.011

[70] Zhou RH, Lee TS, Tsou TC et al Stent implantation activates Akt in the vessel wall: role of mechanical stretch in vascular smooth muscle cells. Arterioscler Thromb Vasc Biol 2003;23:2015–20. 12969991 10.1161/01.ATV.0000095161.06906.ED

[71] Gyöngyösi M, Yang P, Khorsand A et al Longitudinal straightening effect of stents is an additional predictor for major adverse cardiac events. J Am Coll Cardiol 2000;35:1580–9. 10807464 10.1016/s0735-1097(00)00570-2

[72] LaDisa JF Jr., Olson LE, Molthen RC et al Alterations in wall shear stress predict sites of neointimal hyperplasia after stent implantation in rabbit iliac arteries. Am J Physiol Heart Circ Physiol 2005;288:H2465–75. 15653759 10.1152/ajpheart.01107.2004

[73] Moore JE, Berry JL. Fluid and solid mechanical implications of vascular stenting. Ann Biomed Eng 2002;30:498–508. 12086001 10.1114/1.1458594

[74] Benard N, Coisne D, Donal E et al Experimental study of laminar blood flow through an artery treated by a stent implantation: characterisation of intra-stent wall shear stress. J Biomech 2003;36:991–8. 12757808 10.1016/s0021-9290(03)00068-x

[75] Carlier SG, van Damme LC. a, Blommerde CP et al Augmentation of wall shear stress inhibits neointimal hyperplasia after stent implantation: inhibition through reduction of inflammation? Circulation 2003;107:2741–6. 12742998 10.1161/01.CIR.0000066914.95878.6D

[76] Beier S, Ormiston J, Webster M et al Hemodynamics in Idealized Stented Coronary Arteries: important Stent Design Considerations. Ann Biomed Eng 2016;44:315–29. 26178872 10.1007/s10439-015-1387-3 PMC4764643

[77] Kokkalis E, Aristokleous N, Houston JG. Haemodynamics and flow modification stents for peripheral arterial disease: a review. Ann Biomed Eng 2016;44:466–76. 26467554 10.1007/s10439-015-1483-4 PMC4764640

[78] Campbell ID, Humphries MJ. Integrin structure, activation, and interactions. Cold Spring Harb Perspect Biol 2011;3:1–14. 10.1101/cshperspect.a004994 PMC3039929 21421922

[79] Schüller-Ravoo S, Zant E, Feijen J et al Preparation of a designed poly(trimethylene carbonate) microvascular network by stereolithography. Adv Healthc Mater 2014;3:2004–11. 25319598 10.1002/adhm.201400363

[80] Migliavacca F, Petrini L, Montanari V et al A predictive study of the mechanical behaviour of coronary stents by computer modelling. Med Eng Phys 2005;27:13–8. 15604000 10.1016/j.medengphy.2004.08.012

[81] Uthamaraj S, Tefft BJ, Klabusay M et al Design and validation of a novel ferromagnetic bare metal stent capable of capturing and retaining endothelial cells. Ann Biomed Eng 2014;42:2416–24. 25138164 10.1007/s10439-014-1088-3

[82] Wang C, Tian Z, Liu J et al Hemodynamic alterations after stent implantation in 15 cases of intracranial aneurysm. Acta Neurochir (Wien) 2016;158:811–9. 26746828 10.1007/s00701-015-2696-x PMC4918465

[83] Kang SJ, Kim WJ, Lee JY et al Hemodynamic impact of changes in bifurcation geometry after single-stent cross-over technique assessed by intravascular ultrasound and fractional flow reserve. Catheter Cardiovasc Interv 2013;82:1075–82. 23592548 10.1002/ccd.24956

[84] Timmins LH, Meyer CA, Moreno MR et al Effects of stent design and atherosclerotic plaque composition on arterial wall biomechanics. J Endovasc Ther 2008;15:643–54. 19090628 10.1583/08-2443.1 PMC2793418

[85] Nienaber CA, Kische S, Rousseau H et al Endovascular repair of type B aortic dissection: long-term results of the randomized investigation of stent grafts in aortic dissection trial. Circ Cardiovasc Interv 2013;6:407–16. 23922146 10.1161/CIRCINTERVENTIONS.113.000463

[86] Brugaletta S, Gogas BD, Garcia-Garcia HM et al Vascular compliance changes of the coronary vessel wall after bioresorbable vascular scaffold implantation in the treated and adjacent segments. Circ J 2012;76:1616–23. 22531596 10.1253/circj.cj-11-1416

[87] Huang QH, Wu YF, Xu Y et al Vascular geometry change because of endovascular stent placement for anterior communicating artery aneurysms. AJNR Am J Neuroradiol 2011;32:1721–5. 21816920 10.3174/ajnr.A2597 PMC7965400

[88] Dörler J, Frick M, Hilber M et al Coronary stents cause high velocity fluctuation with a flow acceleration and flow reduction in jailed branches: an in vitro study using laser-Doppler anemometry. Biorheology 2012;49:329–40. 23380899 10.3233/BIR-2012-0617

[89] Oz AA, Oz AZ, Arici S. In-vitro bond strengths and clinical failure rates of metal brackets bonded with different light-emitting diode units and curing times. Am J Orthod Dentofac Orthop 2016;149:212–6. 10.1016/j.ajodo.2015.07.036 26827977

[90] Gomez S, Vlad MD, Lopez J et al Design and properties of 3D scaffolds for bone tissue engineering. Acta Biomater 2016;42:341–50. 27370904 10.1016/j.actbio.2016.06.032

[91] Berry JL, Santamarina A, Moore JE et al Experimental and computational flow evaluation of coronary stents. Ann Biomed Eng 2000;28:386–98. 10870895 10.1114/1.276

[92] Luscher TF, Steffel J, Eberli FR et al Drug-eluting stent and coronary thrombosis – biological mechanisms and clinical implications. Circulation 2007;115:1051–8. 17325255 10.1161/CIRCULATIONAHA.106.675934

[93] Makino A, Shin HY, Komai Y et al Mechanotransduction in leukocyte activation: a review. Biorheology 2007;44:221–49. 18094448

[94] Woolf E, Grigorova I, Sagiv A et al Lymph node chemokines promote sustained T lymphocyte motility without triggering stable integrin adhesiveness in the absence of shear forces. Nat Immunol 2007;8:1076–85. 17721537 10.1038/ni1499

[95] Skowasch D, Jabs A, Andrié R et al Presence of bone-marrow- and neural-crest-derived cells in intimal hyperplasia at the time of clinical in-stent restenosis. Cardiovasc Res 2003;60:684–91. 14659814 10.1016/j.cardiores.2003.09.001

[96] Bauriedel G, Jabs A, Kraemer S et al Neointimal expression of rapamycin receptor FK506-binding protein FKBP12: postinjury animal and human in-stent restenosis tissue characteristics. J Vasc Res 2008;45:173–8. 17962721 10.1159/000110417

[97] Tuleta I, Skowasch D, Peuster M et al Cells of primarily extravascular origin in neointima formation following stent implantation. Cardiology 2008;110:199–205. 18057885 10.1159/000111930

[98] Cui X, Zhang X, Guan X et al Shear stress augments the endothelial cell differentiation marker expression in late EPCs by upregulating integrins. Biochem Biophys Res Commun 2012;425:419–25. 22846566 10.1016/j.bbrc.2012.07.115

